# A comparative study of AI chatbots and traditional medical sources for hysterectomy patient education: Assessing professionalism, readability, and patient education quality

**DOI:** 10.1097/MD.0000000000044403

**Published:** 2025-09-12

**Authors:** Guanghua Zhou, Yang Wang, Xiangming Che

**Affiliations:** a Department of Anesthesiology, Beijing Obstetrics and Gynecology Hospital, Capital Medical University; Beijing Maternal and Child Health Care Hospital, Beijing, China.

**Keywords:** AI chatbots, ChatGPT, gemini, generative artificial intelligence, healthcare communication, hysterectomy, medical information quality, patient education, PEMAT score, professionalism in AI, readability assessment

## Abstract

This study compared professionalism, readability, and patient education quality between artificial intelligence (AI)-generated responses (ChatGPT and Gemini) and the American Society of Anesthesiologists (ASA) website for 8 frequently asked questions. To compare the differences in professionalism, readability, and patient education quality between AI (ChatGPT and Gemini) and the ASA website when answering 8 common hysterectomy questions, and to assess whether AI-generated content can serve as a reliable source of patient education for hysterectomy. Blinded experts evaluated professionalism, while 6 readability indices and the patient education materials assessment tool were used to assess content quality. Statistical comparisons were performed with *P* <.05 considered significant. ChatGPT and Gemini demonstrated significantly higher professionalism scores than the ASA website (*P* <.05); however, their readability was lower (*P* <.05). There were no significant differences in professionalism or readability between the ChatGPT and Gemini (*P* >.05). Although AI responses align with clinical guidelines, their low readability poses a usability concern. AI-driven content provides professional and accurate patient education on hysterectomy. However, further refinements are required to improve accessibility without compromising quality.

## 1. Introduction

Generative artificial intelligence (AI) (e.g., ChatGPT and Gemini) is revolutionizing healthcare, including patient education, clinical decision support, and medical research.^[1]^ Models such as ChatGPT (OpenAI) and Gemini Pro (Google DeepMind) use large-scale datasets to generate human-like text, positioning them as potential alternatives to traditional medical resources.^[2]^ However, the quality, readability, and usability of AI-generated medical information, particularly when compared with established sources such as the American Society of Anesthesiologists (ASA) website, remain under investigation.^[3]^ Several studies have examined the performance of AI across diverse medical fields, highlighting the potential limitations of these models. For instance, ChatGPT has demonstrated high accuracy and completeness in responding to cancer-related patient queries, and has even surpassed human candidates in objective structured clinical examinations. Despite these advances, concerns regarding text readability and contextual relevance persist in the clinical setting. Comparisons with traditional resources, including National Comprehensive Cancer Network (NCCN) guidelines and patient-oriented videos, underscore the importance of ensuring accessible and actionable communication in clinical settings. In the context of hysterectomy, a procedure associated with substantial clinical and emotional considerations, effective patient education is vital to support informed decision-making and optimize care outcomes. Although ASA-endorsed resources provide concise and reliable medical information, As AI tools gain traction, rigorous assessment of their professional accuracy, readability^[4]^ and usability in hysterectomy education is critical.

Accordingly, this study examined whether generative AI models, specifically ChatGPT (OpenAI) and Gemini Pro (Google DeepMind), can function as effective and reliable resources for patient education on hysterectomy frequently asked questions (FAQs) by comparing their professional quality with ASA content, analyzing readability through validated metrics (e.g., Flesch Reading Ease and Gunning Fog Index), and evaluating understandability and actionability using the Patient Education Materials Assessment Tool (PEMAT). This study aims to define AI’s value and limitations of AI in clinical practice while identifying opportunities for improvement.

## 2. Methods

### 2.1. Study design

Eight FAQs related to hysterectomy were selected from the American Society of Anesthesiology (ASA) website.^[5]^ These questions covered key topics such as preoperative preparation, anesthesia options, postoperative recovery, and surgical techniques. To generate AI responses, each question was input into ChatGPT 4.0 and Gemini Pro 1.5 in isolated dialogue sessions to prevent cross-learning.

ASA responses and AI-generated content were compiled into a standardized document for expert evaluation. This approach enables a side-by-side comparison of AI-generated content with well-established medical resources (i.e., ASA).

### 2.2. Text evaluation

Three primary domains were used to assess responses: professionalism, readability, and patient education quality. All evaluations were performed independently by designated experts to minimize bias and ensure scoring consistency.

#### 2.2.1. *Professionalism assessment*

Each response was independently evaluated by 2 anesthesiology experts and 2 gynecology experts. The scoring process focused on 3 dimensions: scientific accuracy and guideline consistency (40 points), comprehensiveness (30 points), and logical coherence and practicality (30 points), with higher scores indicating stronger alignment with current medical standards. Scores were weighted according to each question’s primary focus (anesthesia vs gynecology). For anesthesia-related queries (Q4, Q5, Q6, and Q8), anesthesiology experts contributed 70% of the score, and gynecology experts contributed 30%, whereas the reverse proportions were used for gynecology-related queries (Q1, Q2, Q3, and Q7). Cohen kappa was calculated to assess inter-rater reliability and ensure consistency among the reviewers.

#### 2.2.2. *Readability analysis*

The readability of AI-generated and ASA-authored responses was evaluated using 6 standardized metrics: Flesch-Kincaid Grade Level, Gunning Fog Index, SMOG Index, Coleman-Liau Index, Flesch Reading Ease, and automated readability index. These indices quantitatively measured text complexity and ease of understanding, allowing a direct comparison between AI-generated content and the traditional medical resources provided by ASA555.

#### 2.2.3. Patient education quality assessment

The PEMAT was used to evaluate the effectiveness of the materials to cater to patient needs. This tool examines understandability (how clearly information is communicated) and actionability (how well it facilitates informed decision-making). Discrepancies among reviewers were resolved by a fifth obstetric anesthesiologist.

### 2.3. Independent evaluations

All evaluations were performed blindly to minimize bias. The responses were anonymized, randomized, and labeled with unique identifiers, so that the reviewers could not distinguish their sources. A standardized scoring rubric was provided to each expert to maintain uniformity in the evaluation criteria, thereby enhancing the reliability of the comparative analysis.

### 2.4. Statistical analysis

All data were analyzed using statistical methods. One-way analysis of variance (ANOVA) was performed to compare professionalism, readability, and patient education quality scores among the different groups. If a significant difference was detected (*P* <.05), a Bonferroni post hoc test was conducted. Cohen kappa coefficient was used to assess the inter-rater reliability between the 2 anesthesiology experts and gynecology experts. Continuous variables are presented as mean ± standard deviation (mean ± SD), while categorical variables are expressed as frequencies and percentages (%). All statistical tests were 2-tailed and the significance level was set at *P* <.05.

### 2.5. Ethical considerations

This study analyzed publicly available medical resources, including AI content and ASA materials. No personal patient information, clinical data, or health records were included; therefore, ethical committee approval was not required. To ensure scientific rigor and impartiality, all expert evaluations were conducted in a blinded manner to minimize potential bias. This study adhered to the ethical principles outlined in the Declaration of Helsinki and followed the established research guidelines for medical AI applications.

## 3. Results

### 3.1. Professionalism scores

The final weighted professionalism scores for the ChatGPT, Gemini, and ASA are presented in Table 1. Gemini scored highest on professionalism (92.95), followed by ChatGPT (90.35), and ASA (86.15). One-way ANOVA indicated a statistically significant difference among the 3 groups (F[2,6] = 9.85, *P* = .005). post hoc paired *t*-tests revealed that both ChatGPT and Gemini significantly outperformed ASA (*P* <.05), whereas the difference between ChatGPT and Gemini was not statistically significant (*P* >.05).

**Table 1:** Professionalism scores and readability metrics.

Metric	Model	Score	ANOVA (*P*)	ChatGPT versus ASA (*P*)	Gemini versus ASA (*P*)	ChatGPT versus Gemini (*P*)
Professionalism	ChatGPT	90.35	.005*	.031*	–	.09**
	ASA	86.15	–	–	–	–
	Gemini	92.95	–	–	.018*	–
Readability: Average Reading Level	ChatGPT	13.31	.004*	.007*	.002*	.07**
	ASA	11.87	–	–	–	–
	Gemini	13.47	–	–	.001 a	–
Readability: Flesch Reading Ease	ChatGPT	30.88	.001*	.005**	.002*	.07**
	ASA	39.38	–	–	–	–
	Gemini	32.00	–	–	.002*	–
Readability: Gunning Fog Index	ChatGPT	14.08	.011*	.013*	.003*	.18**
	ASA	12.58	–	–	–	–
	Gemini	13.80	–	–	.003*	–
Readability: SMOG Index	ChatGPT	9.99	.014*	.014*	.004*	.16**
	ASA	10.33	–	–	–	–
	Gemini	10.74	–	–	.003 a	–

ANOVA = analysis of variance, ASA = American Society of Anesthesiologists, SMOG Index = Simple Measure of Gobbledygook Index.

*P* <.05 indicates statistical significance.

* *P* < .05, statistically significant.

** *P* >.05, not statistically significant.

### 3.2. Readability analysis

The readability indices for ChatGPT, Gemini, and ASA are summarized in Table 1. ANOVA revealed significant differences (*P* <.05) among the 3 sources for multiple readability metrics.

#### 3.2.1. ASA content

Demonstrated the greatest readability, as reflected by a higher Flesch Reading Ease score (39.38) than ChatGPT (30.88) and Gemini (32.00).

#### 3.2.2. AI-generated responses

AI-generated text showed higher complexity (Flesch-Kincaid, Gunning Fog, SMOG indices) than the ASA content.

#### 3.2.3. Comparison between AI models

There was no statistically significant difference (*P* >.05) between ChatGPT and Gemini on any readability measure, suggesting comparable levels of text complexity.

### 3.3. PEMAT scores

Table 2 presents the PEMAT scores for understandability and actionability across the ChatGPT, Gemini, and ASA.

**Table 2:** PEMAT understandability and actionability scores.

Comparison	Understanding Mean ± SD	Understanding *T*-statistic	Understanding *P*-value	Operability Mean ± SD	Operability T-statistic	Operability *P*-value
ChatGPT versus ASA	0.74 ± 0.05 versus 0.73 ± 0.06	1.61	.12	0.58 ± 0.07 versus 0.54 ± 0.07	4.26	*P* <.001*
ChatGPT versus Gemini	0.74 ± 0.05 versus 0.76 ± 0.04	−1.14	.26	0.58 ± 0.07 versus 0.57 ± 0.06	0.78	.44
ASA versus Gemini	0.73 ± 0.06 versus 0.76 ± 0.04	−4.45	*P* <.001*	0.54 ± 0.07 versus 0.57 ± 0.06	−3.1	*P* <.001*

ASA = American Society of Anesthesiologists, PEMAT = Patient Education Materials Assessment Tool, SD = standard deviation.

*P* <.05 indicates statistical significance.

* *P* < .05, statistically significant.

#### 3.3.1. Gemini’s understandability

Score was the highest (0.76 ± 0.04), significantly surpassing ASA (0.73 ± 0.06, *P* < .001).

#### 3.3.2. ChatGPT’s understandability

Achieved 0.74 ± 0.05, showing no statistically significant difference from ASA (*P* = .12) or Gemini (*P* = .26).

#### 3.3.3. Actionability across AI models

ChatGPT (0.58 ± 0.07) and Gemini (0.57 ± 0.06) both exceeded ASA (0.54 ± 0.07) in actionability (*P* < .001 for ChatGPT and *P* = .002 for Gemini). No significant difference was observed between the AI models (*P* = .44).

Taken together, Gemini provided the highest overall understandability, whereas ChatGPT provided actionability. Both AI-based responses outperformed ASA content in multiple dimensions of patient education quality, underscoring their potential value in clinical practice.

## 4. Discussion

This study evaluated the performance of the ChatGPT, Gemini, and ASA websites in delivering information about hysterectomy. The assessment encompassed professionalism, readability, and quality of patient education, as measured by the PEMAT scores. The results highlight the advantages and drawbacks of AI-generated medical content compared to well-established professional sources.

### 4.1. Professionalism

Both AI models outperformed ASA in terms of scientific accuracy, guideline adherence, comprehensiveness, and logical coherence. This suggests that AI-generated responses are not only thorough, but also generally aligned with current clinical guidelines. Among the 2 AI models, Gemini attained the highest professionalism score, although the difference from ChatGPT was not statistically significant (*P* >.05).

This may reflect AI’s ability of AI to synthesize vast, current medical datasets from diverse sources. However, these models lack transparent citation systems, which hinders their ability to verify referenced guidelines or studies. As a result, although AI-generated content may be comprehensive, the absence of direct citations limits its reliability in clinical practice.

### 4.2. Readability

The ASA content was notably more readable than either ChatGPT or Gemini. Both AI models generated relatively complex text, as evidenced by the lower Flesch Reading Ease scores and higher Flesch-Kincaid and Gunning Fog indices. The AI’s higher reading level (above 8th grade^[6]^) may limit accessibility for diverse patient populations. Future developments in AI-driven medical resources should emphasize language simplification, while preserving scientific accuracy, to ensure greater accessibility.

### 4.3. Patient education quality

In the PEMAT assessment, Gemini attained the highest understandability score, indicating that its content was more comprehensible than that of ASA and ChatGPT. Conversely, the ChatGPT achieved the highest actionability score, suggesting that the information provided was especially conducive to informed patient decision-making. Gemini’s higher understandability and ChatGPT’s actionability highlight AI’s multifaceted value of AI in patient education.

### 4.4. Clinical implications

The findings of this study bear a significant weight for the integration of AI technologies into patient education. Credibility and source attribution remain key concerns given that AI-generated information, although often high in quality, lacks transparent citation mechanisms, making verification difficult. Improving citation traceability can enhance clinical acceptance of AI content.

In addition, readability optimization is critical for ensuring that individuals with varied health literacy can effectively engage with AI-generated materials.^[7]^ Simplifying the language without compromising scientific accuracy should be a priority for future AI model development.

AI chatbots may play a complementary role by supplementing established resources such as the ASA website by offering personalized and detailed explanations. Nevertheless, expert oversight is crucial for maintaining quality and accuracy. Finally, regulatory and professional bodies should be actively involved in validating AI-based medical content prior to its widespread adoption, particularly in patient education contexts.

## 5. Conclusion

ChatGPT and Gemini have demonstrated substantial promise for generating accurate and comprehensive medical information. Nonetheless, improving readability is essential to maximize the utility of patient education. Expert-reviewed AI systems have the potential to serve as valuable adjuncts to traditional medical resources, provided that they undergo continuous validation and maintain rigorous standards of transparency and accountability.

## Author contributions

**Conceptualization:** Guanghua Zhou, Xiangming Che.

**Data curation:** Guanghua Zhou.

**Formal analysis:** Guanghua Zhou, Yang Wang.

**Investigation:** Guanghua Zhou, Yang Wang.

**Methodology:** Guanghua Zhou.

**Project administration:** Guanghua Zhou.

**Resources:** Guanghua Zhou.

**Software:** Guanghua Zhou.

**Supervision:** Xiangming Che.

**Validation:** Xiangming Che.

**Visualization:** Guanghua Zhou, Yang Wang.

**Writing – original draft:** Guanghua Zhou.

**Writing – review & editing:** Guanghua Zhou, Yang Wang.
